# Supplementary material for: Descriptions of Three New Species of the Genus Acerataspis Uchida, 1934 (Hymenoptera, Ichneumonidae, Metopiinae), with an Illustrated Identification Key to Extant Species
Source: Insects. 2023 Apr 17;14(4):389. doi: 10.3390/insects14040389 (PMC10142930; doi:10.3390/insects14040389)
Supplement: Supplementary file 1 [file insects-14-00389-s001.zip › Table S1.pdf]

Revision of the World Species of the Genus *Acerataspis* (Hymenoptera, Ichneumonidae, Metopiinae)

Jing-Xian Liu [1], Alexey Reshchikov [2] and Hua-Yan Chen [3,*]

1. Department of Entomology, South China Agricultural University, Guangzhou 510642, China

2. Institute of Eastern Himalaya Biodiversity Research, Dali University, Dali 671003, China

3 Key Laboratory of Plant Resources Conservation and Sustainable Utilization, South China Botanical Garden, Chinese Academy of Sciences, Guangzhou 510650, China

* Correspondence: huayanc@scbg.ac.cn

Supplements

Table S1. Interspecific pairwise distance of *Acerataspis* based on *COI* sequences (%)

	species	1	2	3	4	5	6 0
1	*Acerataspis clavata*		13.2–15	11.4–12.8	12.9–13.4	9.8–10.7	12.1–13.3
2	*Acerataspis fukienensis*			13.6–14.1	14.2–14.6	14.6–15.7	16.4–16.5
3	*Acerataspis fusiformis*				13.9–14.4	11.1–12.4	9.7–10.4
4	*Acerataspis maliae* sp. n.					12.3–12.7	15–15.3
5	*Acerataspis separata* sp. n.						12.7–13
6	*Acerataspis similis* sp. n.						
