# Supplementary material for: Descriptions of Three New Species of the Genus Acerataspis Uchida, 1934 (Hymenoptera, Ichneumonidae, Metopiinae), with an Illustrated Identification Key to Extant Species
Source: Insects. 2023 Apr 17;14(4):389. doi: 10.3390/insects14040389 (PMC10142930; doi:10.3390/insects14040389)
Supplement: Supplementary file 1 [file insects-14-00389-s001.zip › Table S2 Genetic distance of COI within species under K2P model.pdf]

Revision of the Genus *Acerataspis* from Asia (Hymenoptera, Ichneumonidae, Metopiinae)

Jing-Xian Liu [1], Alexey Reshchikov [2] and Hua-Yan Chen [3,*]

1. Department of Entomology, South China Agricultural University, Guangzhou 510642, China

2. Institute of Eastern Himalaya Biodiversity Research, Dali University, Dali 671003, China

3. Key Laboratory of Plant Resources Conservation and Sustainable Utilization, South China Botanical Garden, Chinese Academy of Sciences, Guangzhou 510650, China

* Correspondence: huayanc@scbg.ac.cn

Supplements

Table S2 Genetic distance of *COI* within species under K2P model

Species	Distance (%)
Acerataspis clavata	0-3.6
Acerataspis fukienensis	0.3
Acerataspis fusiformis	0-2.2
Acerataspis maliae sp. n.	0.3
Acerataspis separata sp. n.	0-3.9
Acerataspis similis sp. n.	NA
